# Photocatalytic Formic Acid Conversion on CdS Nanocrystals with Controllable Selectivity for H_2_ or CO**

**DOI:** 10.1002/anie.201502773

**Published:** 2015-07-16

**Authors:** Moritz F Kuehnel, David W Wakerley, Katherine L Orchard, Erwin Reisner

**Affiliations:** Christian Doppler Laboratory for Sustainable SynGas Chemistry, Department of Chemistry, University of Cambridge Lensfield Road, CB2 1EW, Cambridge (UK) E-mail: http://www-reisner.ch.cam.ac.uk Homepage: http://www-reisner.ch.cam.ac.uk

**Keywords:** CdS, formic acid, hydrogen, photocatalysis, quantum dots

## Abstract

Formic acid is considered a promising energy carrier and hydrogen storage material for a carbon-neutral economy. We present an inexpensive system for the selective room-temperature photocatalytic conversion of formic acid into either hydrogen or carbon monoxide. Under visible-light irradiation (*λ*>420 nm, 1 sun), suspensions of ligand-capped cadmium sulfide nanocrystals in formic acid/sodium formate release up to 116±14 mmol H_2_ g_cat_^−1^ h^−1^ with >99 % selectivity when combined with a cobalt co-catalyst; the quantum yield at *λ*=460 nm was 21.2±2.7 %. In the absence of capping ligands, suspensions of the same photocatalyst in aqueous sodium formate generate up to 102±13 mmol CO g_cat_^−1^ h^−1^ with >95 % selectivity and 19.7±2.7 % quantum yield. H_2_ and CO production was sustained for more than one week with turnover numbers greater than 6×10^5^ and 3×10^6^, respectively.

The replacement of conventional fossil fuels with a CO_2_-neutral energy cycle is a key global challenge for developing a sustainable economy. Hydrogen holds promise as a secondary energy vector for use in fuel cells, but its safe storage and transport remain the subject of intensive research.[1] Formic acid (HCO_2_H, FA) has received considerable attention as a potential renewable fuel of high energy density. Its low toxicity and high gravimetric hydrogen content of 4.4 % render FA a promising hydrogen storage material, with CO_2_ as the only by-product of H_2_ release.[2] CO_2_ recycling by mild homogeneous hydrogenation of CO_2_ to FA has become a feasible process to store H_2_ derived from renewable sources.[3] In addition, a growing number of synthetic catalysts[4] and enzymes[5] promote the storage of electrical energy by electrochemical reduction of CO_2_ to FA. FA is also a major product of biomass processing.[6]

Although FA dehydrogenation (FA-to-H_2_) is an exergonic process (Scheme 1), efficient liberation of H_2_ requires additional energy input (i.e. high temperatures or light) unless precious-metal-based catalysts are employed.[7, 8] The high cost and low abundance of these catalysts, however, precludes scalability and thus a widespread application. Precious-metal-free alternatives typically require elevated temperatures and organic solvents, limiting their potential use in portable applications and decreasing their overall energy density.[9] Only recently, the photochemical decomposition of FA has attracted increasing interest as an alternative approach to generate H_2_ from FA at room temperature. Photocatalysts based on Pd,[10] AuPd,[11] Pt,[12] Rh,[13] and Ru[14] have demonstrated activities up to 154 mmol H_2_ g_cat_^−1^ h^−1^. Examples of precious-metal-free catalysts are scarce and show substantially lower activity.[15]

**Scheme 1:**
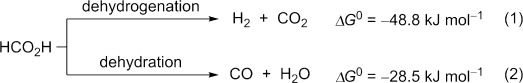
Thermodynamics of formic acid decomposition pathways.[16]

Despite the pivotal role of nanocrystalline semiconductors (commonly referred to as quantum dots, QDs)[17] in photovoltaics[18] and artificial photosynthesis,[19] little is known about their activity towards photochemical FA-to-H_2_ conversion. Cadmium sulfide is among the most studied QD materials owing to its ease of preparation, low cost, and high absorption of visible light. Whereas bulk CdS powder shows limited photocatalytic FA-to-H_2_ activity,[20] enhanced activity has been achieved by confinement on a titanate nanotube support,[21] by construction of CdS-ZnS heterojunctions,[22] and by introduction of precious-metal co-catalysts such as Pt[21a, 23] and Ru.[22, 24] A precious-metal-free hybrid system comprised of CdS and a H_2_-producing enzyme (hydrogenase) exhibited low selectivity and suffered from enzyme inhibition.[25] Moreover, none of these systems show flexibility with respect to the reaction products.

FA is known to undergo two pathways of decomposition, to give either H_2_ or carbon monoxide (Scheme 1). CO is a valuable synthon in the chemical industry and synthesis gas (a mixture of CO and H_2_) can be used to generate liquid fuels such as methanol and hydrocarbons by the Fischer–Tropsch process.[26] CO is currently produced from fossil sources, but despite its critical importance, the sustainable generation of CO from FA has received little attention, and no chemical storage process for CO is currently available.[27] FA contains more than 60 wt % CO, which can be released upon treatment with excess dehydrating agents, such as conc. H_2_SO_4_, but catalytic FA-to-CO conversion typically requires high temperatures.[28] Consequently, selective photocatalytic FA-to-CO has not been reported. This work presents an inexpensive and highly active CdS-based photocatalyst that efficiently uses FA as a clean storage material for the controlled generation of either H_2_ or CO under ambient conditions.

Monodisperse CdS nanocrystals with 3-mercaptopropionic acid (MPA) capping ligands (QD-MPA) were prepared according to literature procedures from oleic acid capped CdS nanoparticles by basic ligand exchange (*λ*_max_=443 nm, *D*=4.4±0.4 nm, Figure S1).[19c, 29] Under simulated solar light irradiation (AM1.5G, 100 mW cm^−2^, *λ*>420 nm, 25 °C, see Supporting Information for details), dispersions of QD-MPA in 4.0 m sodium formate in FA (Laboratory Reagent Grade, >90 %) generated H_2_ at initial rates of 52.1±6.6 mmol H_2_ g_cat_^−1^ h^−1^ (Figure 1 A, Table 1). The addition of up to 0.5 mm CoCl_2_⋅6 H_2_O resulted in enhanced rates of up to 116±14 mmol H_2_ g_cat_^−1^ h^−1^ (QD/Co ratio ca. 500:1, see Figure S2 and Tables S1 and S2 for optimization details). Only traces of CO were detected in the headspace gas (670±146 ppm absolute, 0.614±0.065 % with respect to H_2_). When the full solar spectrum was used for irradiation, the rate increased to 218±22 mmol H_2_ g_cat_^−1^ h^−1^ (Figure S3), showing that both the visible and UV portion of the solar spectrum are used for catalysis; a dependence of the catalyst on light was confirmed by varying the light intensity (Figure S4). At *λ*=460 nm, the external quantum yield (EQY) was 21.2±2.7 % (Table S3). The CO_2_/H_2_ ratio increased over time (Figure S5), but remained below the theoretical 1:1 stoichiometry (Scheme 1), presumably because of a higher solubility of CO_2_ over H_2_ in FA. H_2_ generation even proceeds in FA without added sodium formate, albeit at a lower rate of 1.4±0.3 mmol H_2_ g_cat_^−1^ h^−1^. No H_2_ was formed in the dark or without photocatalyst (Table S2).

**Figure 1:**
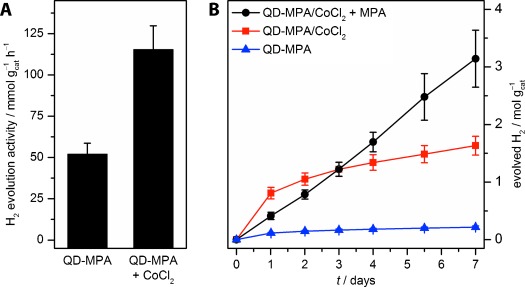
Photocatalytic dehydrogenation of FA: A) Initial activity of QD-MPA with and without added co-catalyst (1 h of irradiation); B) long-term activity of QD-MPA [AM1.5G, 100 mW cm^−2^, *λ*>420 nm; 0.91 μm QD-MPA (176 μg mL^−1^), 4.0 m NaHCO_2_ in FA; CoCl_2_=0.5 mm CoCl_2_⋅6 H_2_O, MPA=140 mm 3-mercaptopropionic acid].

**Table 1:** Comparison of selected photocatalysts for visible-light-driven FA-to-H_2_ conversion under ambient conditions (see Table S5 for more examples)

Catalyst	Activity^[a]^ [mmol H_2_ g_cat_^−1^ h^−1^]	Selectivity^[b]^ [%]	EQY [%]	Lifetime [h]	Ref.
Pd-C_3_N_4_	53.4	100	n/a	>6	[10b]
AuPd-TiO_2_	17.7^[c]^	99.7	15.6	>9	[11b]
Pt-CdS	1.22	n/a	21.4	>30	[23a]
Ru-CdS/ZnS	5.85±0.09	n/a	20	>40	[22]
CdS/ZnS	1.24±0.02	n/a	n/a	>40	[22]
H_2_ase-CdS^[d]^	0.356	20	3.1	>3.5	[25]
[{RuCl_2_(PhH)}_2_] + 12 PPh_3_	154^[e]^	n/a	n/a	>5	[14]
[Fe_3_(CO)_12_] + PPh_3_, tpy^[f]^	2.7^[e]^	“trace CO”	n/a	>24	[15c]
QD-MPA	52.1±6.6	98.8±0.1	n/a	>168	this work
QD-MPA/CoCl_2_	116±14	99.4±0.1	21.2±2.7	>168	this work
QD-MPA/CoCl_2_	218±22^[c]^	98.9±0.1	n/a	>24	this work

[a] For an accurate comparison, published data were converted into gravimetric activity using the mass of the entire photocatalyst used in the reaction. [b] Selectivity=100 %×*n* H_2_/(*n* H_2_+*n* CO). [c] Full solar spectrum irradiation. [d] H_2_ase=hydrogenase. [e] *λ*>380 nm. [f] tpy=2,2′:6′,2′′-terpyridine. n/a=not available.

Long-term experiments were performed to demonstrate the stability of this system (Figure 1 B, Table S4). H_2_ evolution was sustained for more than 7 days, even in the absence of a co-catalyst. The rate of H_2_ evolution gradually decreased during the first 48 h of irradiation, stabilizing at 4.4±1.3 mmol H_2_ g_cat_^−1^ h^−1^ and 0.52±0.17 mmol H_2_ g_cat_^−1^ h^−1^ with and without co-catalyst, respectively. When excess MPA (140 mm) was added to the QD-MPA/CoCl_2_ system prior to irradiation, a lower initial rate of 18.7±2.9 mmol H_2_ g_cat_^−1^ h^−1^ was observed, but this activity remained constant over the course of one week. More than 3 mol H_2_ g_cat_^−1^ were generated after one week, which corresponds to over 600 000 turnovers per QD. Transmission electron microscopy (TEM) after photocatalysis indicated the formation of aggregates that retained nanocrystalline features (Figure S6). Particle aggregation over time was monitored in situ by UV/Vis spectroscopy. When QD-MPA is added to FA, a red-shift of the absorption maximum indicates aggregation, which increases further during irradiation (Figure S7 A).[30] In the presence of MPA, a red-shift was observed upon addition to FA, but no further change occurred during irradiation (Figure S7 B), suggesting that MPA enhances the lifetime of QD-MPA during photocatalysis by preventing aggregation.

To the best of our knowledge, the catalytic activity and lifetime of QD-MPA/CoCl_2_ surpasses all previously reported heterogeneous photocatalysts for FA-to-H_2_ conversion under ambient conditions, including those based on precious metals (Table 1, see Table S5 for a more comprehensive comparison). The most active heterogeneous catalyst to date, a Pd-C_3_N_4_ nanocomposite, evolves 53.4 mmol H_2_ g_cat_^−1^ h^−1^ with quantitative H_2_ selectivity for up to 6 h. Without added co-catalyst, QD-MPA shows a comparable activity, whereas QD-MPA/CoCl_2_ is more than twice as active and shows an improved long-term stability. A Ru-based homogeneous photocatalyst was reported to achieve 154 mmol H_2_ g_cat_^−1^ h^−1^ in DMF solution, but no selectivity was reported.[14] The best precious-metal-free photocatalyst, CdS-ZnS particles, can evolve up to 1.24±0.02 mmol H_2_ g_cat_^−1^ h^−1^ under irradiation with visible light (selectivity not reported),[22] more than two orders of magnitude less than QD-MPA/CoCl_2_.

Insight into the nature of the active catalyst was sought by separating QD-MPA/CoCl_2_ from the reaction mixture by centrifugation after 1 h photocatalysis. When the solid residue was re-dispersed in fresh reaction medium without added co-catalyst, the observed H_2_ evolution activity was similar to QD-MPA in the absence of co-catalyst, suggesting that the active catalyst is not attached to the QDs. The QD-free supernatant did not show any activity (Figure S8, Table S6). Inductively coupled plasma optical emission spectrometry (ICP-OES) measurements of the solid confirm the absence of Co (Co/Cd atomic ratio in the solid less than (4.8±1.9)×10^−4^:1; in contrast to 0.44:1 in the entire sample before catalysis). These findings and the absence of an induction period for H_2_ evolution with QD-MPA/CoCl_2_ lend further support to the homogeneous nature of the active co-catalyst. Cobalt species are known to function as homogeneous hydrogen-evolving catalysts in the presence of nanocrystalline semiconductors.[31] Incorporation of cobalt is also known to enhance the activity of formate oxidation electrocatalysts[32] and there is precedent for homogeneous oxidation of FA by Co^3+^ ions.[33] The mechanism of FA-to-H_2_ conversion could, therefore, consist of formate oxidation by photogenerated holes and subsequent proton reduction by photoexcited electrons.[20, 34] In this case, the reductive half reaction, the oxidative half reaction, or both are enhanced by Co catalysis.

With the reactivity of nanocrystalline CdS towards formate established, we sought possibilities to tune the reaction pathway towards FA-to-CO conversion [Scheme 1, Eq. (2)]. In aqueous formate solution, the activity of QD-MPA was much lower than in FA but showed a reversed selectivity, with CO as the main decomposition product (60±7 % CO, Figure 2 A, Table S2). To enhance the photocatalytic activity in aqueous solution, we studied the effect of modifying the QD surface. Ligand-free, charge-stabilized CdS nanocrystals (QD-BF_4_) were prepared from oleic acid capped CdS nanocrystals by following a modified reactive ligand stripping procedure using [Me_3_O]BF_4_ in the presence of *N*,*N*-dimethylformamide (DMF) to remove the oleic acid capping groups.[35] Ligand stripping led to a blue-shift of the absorption maximum indicative of a decrease in QD size (Figure S9 B). This is presumably a result of etching by BF_4_^−^ ions, as shown for CdTe QDs.[36] Larger QD precursors (*λ*_max_=466 nm, *D*=6.0±0.9 nm) were, therefore, used to obtain QD-BF_4_ with a diameter similar to QD-MPA (*λ*_max_=445 nm, *D*=4.9±0.7 nm, Figure S9). QD-BF_4_ showed a drastically enhanced photocatalytic FA-to-CO activity in water compared to QD-MPA (Figure 2 A). Under optimized conditions, up to 102±13 mmol CO g_cat_^−1^ h^−1^ are formed with 96.3±0.1 % selectivity (4.0 m NaHCO_2_ in 2.5 m KOH/CO_2_ pH 9.7; see Figures S10–S12 and Tables S7 and S8 for optimization details). In FA solution, QD-BF_4_ showed a decomposition selectivity of FA towards H_2_ that was comparable to QD-MPA, but with lower activity (Figure 2 A, Table S2). The selectivity switchover to CO is, therefore, not a result of ligand removal, but promoted by the basic aqueous environment. This is further corroborated by a strong pH-dependence of the product selectivity, with FA-to-H_2_ conversion becoming more pronounced in neutral and acidic solution (Figure S10, Table S7); the addition of MPA to QD-BF_4_ in aqueous formate solution did not affect the product selectivity (see below). The effects of water on the gas-phase photocatalytic FA decomposition have been previously documented.[37]

**Figure 2:**
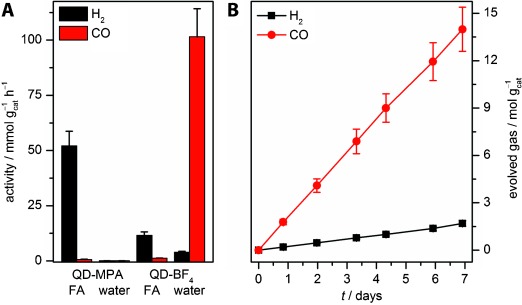
Photocatalytic dehydration of FA (AM1.5G, 100 mW cm^−2^, *λ*>420 nm): A) Effect of solvent on the product selectivity of CdS QDs in the absence of CoCl_2_ co-catalyst (see Table S3 for conditions); B) long term activity of QD-BF_4_ [0.0611 μm QD-BF_4_ (14.4 μg mL^−1^), 4.0 m NaHCO_2_ in 2.5 m aqueous KOH/CO_2_ pH 9.7].

Aqueous QD-BF_4_ is remarkably robust and sustained CO production over the course of one week with no detectable decrease in activity; more than 14 mol CO g_cat_^−1^ were generated, which corresponds to 3 000 000 turnovers per QD (Figure 2 B, Table S9). No activity was seen in the absence of light (Table S2, entry 16; Figure S13), confirming that irradiation provides the necessary activation energy for formate dehydration. Since irradiation of CdS generates electron/hole pairs, we propose that the reaction mechanism is centered around the charge separation by the QD. Assuming that one photon is required to generate one CO molecule, a minimum EQY of 19.7±2.7 % was recorded at *λ*=460 nm (Table S10, see the Supporting Information for further details). Formate was established as the sole source of CO through IR spectroscopy by using ^13^C-labeled sodium formate (Figure S14). TEM analysis after catalysis indicates the formation of nanostructured aggregates (Figure S15). This was corroborated through an observed red-shift in the UV/Vis spectrum (Figure S16, see above).

Addition of CoCl_2_ to QD-BF_4_ or QD-MPA did not increase the photocatalytic activity in aqueous solution (Table S2), indicating that the CdS particle surface itself plays an essential role in the catalytic activity. Mechanistic studies were performed to further support this hypothesis (Tables S11 and S12). Addition of excess Na_2_S to an active sample of QD-BF_4_ in aqueous sodium formate solution resulted in a sudden drop in catalytic activity (Figure 3 A), suggesting that sulfide ions block the catalytically active sites; addition of MPA resulted in a similar effect (Figure 3 B). At the same time, no increase in H_2_ evolution was observed in either case, proving that S^2−^ ions are not simply acting as electron donors and that the addition of MPA has not resulted in a selectivity switch towards H_2_ production. We hypothesize that Cd ions form the active site for CO evolution. A similar decrease in CO evolution activity was observed when EDTA was added to selectively complex surface-bound Cd^2+^, providing further evidence of the crucial role of surface Cd^2+^ ions for dehydration activity. X-ray photoelectron spectra (XPS) of sulfide-poisoned QD-BF_4_ exhibit a slight shift of the Cd(3d) peaks to lower binding energies, very similar to the spectrum of QD-MPA (Figure S18, Table S13). In addition, a shoulder on the Cd(3d) signals, which is absent in unmodified QD-BF_4_, is observed at a lower binding energy. Little effect on the Cd/S stoichiometry was observed by XPS (Table S13). These data indicate that Na_2_S addition leads to a distortion of the Cd environment. The resemblance of the poisoned environment to that of inactive QD-MPA suggests that the active site is Cd-based. Previous studies on thermal FA decomposition have established the importance of surface effects,[38] such as H_2_O coverage[39] and surface acidity/basicity,[40] on the activity and selectivity.

**Figure 3:**
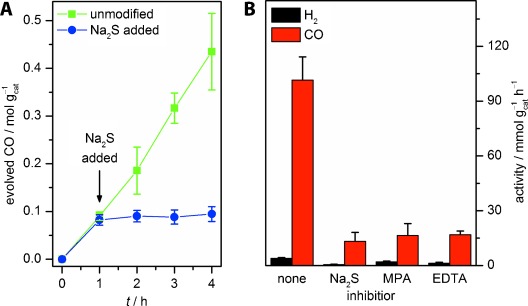
Effects of inhibitors on the photocatalytic dehydration of formate: A) In situ inhibition of photocatalytic activity by Na_2_S addition (111 mm); B) comparison of different inhibitors [100 mW cm^−2^ AM1.5G, *λ*>420 nm; 0.0611 μm QD-BF_4_ (14.4 μg mL^−1^), 4.0 m NaHCO_2_, 2.5 m aqueous KOH/CO_2_ pH 9.7; 111 mm Na_2_S, 111 mm MPA, or 83.3 mm Na_2_EDTA]. EDTA=ethylenediaminetetraacetate.

In summary, we have developed an inexpensive and highly active photocatalyst system for sunlight-driven conversion of formic acid with controlled selectivity and long-term stability. Ligand-capped CdS quantum dots with a cobalt co-catalyst generate H_2_ with unprecedented activity and >99 % selectivity when dispersed in formic acid at room temperature. In contrast, CO formation is strongly favored in aqueous solution after ligand stripping and proceeds with high activity and efficiency. With this first example of selective photocatalytic formic acid to CO conversion, we introduce formic acid as a renewable CO storage material with more than 60 wt % capacity. This work demonstrates that careful matching of engineered particle surfaces with optimized reaction media enables a novel flexibility in the sustainable use of formic acid to generate valuable chemical feedstocks.

## Supporting Information

As a service to our authors and readers, this journal provides supporting information supplied by the authors. Such materials are peer reviewed and may be re-organized for online delivery, but are not copy-edited or typeset. Technical support issues arising from supporting information (other than missing files) should be addressed to the authors.

miscellaneous_information
