# Supplementary material for: Photocatalytic Formic Acid Conversion on CdS Nanocrystals with Controllable Selectivity for H2 or CO
Source: Angew Chem Int Ed Engl. 2015 Jul 16;54(33):9627–31. doi: 10.1002/anie.201502773 (PMC4552973; doi:10.1002/anie.201502773)
Supplement: Supplementary file 1 — miscellaneous_information [file anie0054-9627-sd1.pdf]

Angewandte Chemie
Eine Zeitschrift der Gesellschaft Deutscher Chemiker

Supporting Information

Photocatalytic Formic Acid Conversion on CdS Nanocrystals with Controllable Selectivity for H_2 or CO**

*Moritz F. Kuehnel, David W. Wakerley, Katherine L. Orchard, and Erwin Reisner**

anie_201502773_sm_miscellaneous_information.pdf

Experimental

Reagents. All chemical reagents were obtained from commercial suppliers at the highest available purity for analytical measurements. Formic acid was laboratory reagent (LR) grade (>90%) and $NaH^{13}CO_2$ was purchased from Sigma-Aldrich. 4.0 M sodium formate solutions in formic acid was prepared by dissolving solid $NaHCO_2$ (6.80 g) in formic acid to give a total volume of 25 mL. All solvents were HPLC grade and distilled water was used in all photocatalysis experiments.

QD-MPA. CdS QD capped with oleic acid ligands (QD-OA, λ_{max} = 440 nm, D = 4.5±0.5 nm) were prepared according to a previously reported procedure.[1] A mixture of CdO (0.64 g) and oleic acid (26 g) in octadecene (ODE, 70 g) was heated under an Ar atmosphere to 280 °C. Sulfur (0.08 g) in ODE (30 g) was added rapidly and the solution allowed to cool to 250 °C. This temperature was maintained for 120 s before quenching by rapid cooling. The particles were precipitated from 1:1 hexane:methanol using excess acetone, centrifuged at 7000 rpm for 3 min, and re-dispersed in hexane. Two further washing steps were carried out using hexane and acetone as solvent and non-solvent, respectively, before finally dispersing in hexane. Ligand exchange with 3-mercaptopropionic acid (MPA) was carried out according to a literature procedure.[2] MPA (0.5 mL) was dispersed in 1:1 chloroform:methanol (10 mL) and the pH adjusted to 11 with tetramethyl ammonium hydroxide. QD-OA solution (2 mL) was added to this mixture and stirred in the dark for two days. The QDs were precipitated with excess acetone and centrifuged (7000 RPM, 3 min). The isolated particles were washed with acetone before being dispersed in water. The molar and gravimetric concentrations were determined from UV-vis spectroscopy based on the position and absorbance of the absorption maximum at λ_{max} = 443 nm, corresponding to a diameter of 5.0 nm.[3] TEM analysis was in agreement with a diameter of 4.4±0.4 nm.

QD-BF_4. To prepare ligand-free CdS quantum dots (QD-BF_4) with an absorption maximum equivalent to that of QD-MPA, larger oleic acid capped CdS particles were used as the starting material (λ_{max} = 466 nm, D = 6.0±0.9 nm) in order to compensate for the known etching of the particles during ligand stripping. Both synthesis[1-2] and stripping[4] procedures were modifications of literature procedures. A mixture of CdO (0.64 g) and oleic acid (26 g) in octadecene (ODE, 70 g) was heated under an Ar atmosphere to 280°C. Separately, a solution of sulfur (0.08 g) in ODE (30 g) was prepared. Half of the sulfur solution was added rapidly and the solution allowed to cool to 250 °C. The remaining half of the sulfur solution was added dropwise over 2 min, after which the reaction was quenched by rapid cooling. The particles were precipitated from 1:1 hexane:methanol using excess acetone, centrifuged at 5000 RPM for 3 min, and re-dispersed in hexane. Two further washing steps were carried out using hexane and acetone as solvent and non-solvent, respectively, before finally dispersing in hexane. The resulting QD-OA solution in hexane (3 mL) was reduced to dryness and, under a N_2 atmosphere, re-dispersed in a mixture of anhydrous $CHCl_3$ (9 mL), anhydrous CH_2Cl_2 (6 mL) and anhydrous N,N-dimethylformamide (DMF, 0.9 mL). Aliquots of stripping agent (Me_3OBF_4, 1.0 M in acetonitrile) were added slowly until the particles precipitated. The precipitate was centrifuged (7000 RPM, 3 min), dried in air for 1 min, and re-dispersed in DMF (1–2 mL). The molar and gravimetric concentrations were determined from UV-vis spectroscopy based on the position and absorbance of the absorption maximum at λ_{max} = 445 nm, corresponding to a diameter of 5.1 nm.[3] TEM analysis was in good agreement with a diameter of 4.9±0.7 nm.

Photocatalytic generation of H_2. An aqueous QD-MPA stock solution (215 µM = 41.3 mg mL^{-1}, 8.08 µL), was added to a photoreactor containing 2 mL of a solution of $NaHCO_2$ (4.0 M) and $CoCl_2 \cdot 6H_2O$ (0.5 mM) in formic acid and a magnetic stirrer bar. For control experiments, an aqueous suspension of bulk CdS (25.8 mg mL^{-1}, 5.38 µL) was used instead of QD-MPA. The photoreactor was sealed with a rubber septum and purged with N_2 (2% CH_4) for 10 min in the dark. The photoreactor was then thermostated with a water circulator at 25°C, stirred at 600 RPM and irradiated by a solar light simulator (Newport Oriel, 100 mW cm^{-2}) equipped with an air mass 1.5 global filter (AM1.5G). IR irradiation was filtered with a water filter (10 cm path length) and UV irradiation with a 420 nm cut-off filter (UQG Optics). Product distribution was quantified through periodic headspace gas analysis (30 µL) by gas chromatography.

Centrifugation experiments. After 1 h photocatalytic H_2 production, the reaction mixture was transferred to a centrifuge tube and centrifuged for 10 min at 10,000 RPM. The supernatant was decanted and the pellet was re-suspended in fresh $NaHCO_2$ (4.0 M) in formic acid. For controls, the pellet was re-suspended in the same supernatant to account for losses during transfer to and from the centrifugation tube; in another control experiment, the supernatant was filtered with a 10 µm syringe filter and used for photocatalytic experiments. The resulting suspensions/solutions were transferred to a clean photoreactor which was sealed with a rubber septum and purged with N_2 (2% CH_4) for 10 min in the dark. The photoreactor was irradiated by a solar light simulator at 25°C (*vide supra*).

Photocatalytic generation of CO. A QD-BF_4 stock solution in DMF (10.2 µM = 2.28 mg mL^{-1}, 10.76 µL) was added to a photoreactor containing a magnetic stirrer bar and the DMF was removed *in vacuo.* For control experiments, a suspension of bulk CdS in DMF (10.4 mg mL^{-1}, 2.67 µL) was used instead of QD-BF_4. 2 mL of an aqueous solution of KOH (2.5 M) and $NaHCO_2$ (4.0 M) was added. The photoreactor was sealed with a rubber septum and the solution was purged with CO_2 (2% CH_4) for 10 min in the dark to give a $KHCO_3/K_2CO_3/CO_2$ buffer at pH 9.7 (denoted KOH/CO_2 in the text). The photoreactor was by a solar light simulator at 25°C (*vide supra*). Sodium formate as the sole source of CO was confirmed by using isotopically labelled sodium formate (see below and Figure S14).

Inhibition experiments. After 1 h photocatalytic CO production, MPA (25 µL, 30.5 mg, 290 µmol) or Na_2S (250 µL, 1 M aqueous solution) was added with a microsyringe. Due to its limited solubility, EDTA inhibition was studied by adding 2 mL of an aqueous solution of KOH (2.5 M), $NaHCO_2$ (4.0 M) and $Na_2EDTA \cdot 2H_2O$ (83.3 mM) to a vial containing dried QD-BF_4 followed by a similar sample preparation (*vide supra*).

Gas Chromatography Analysis. Gas chromatography was carried out on an Agilent 7890A gas chromatograph. H_2 was analysed using a HP-5 column (0.32 mm diameter) at 45°C and N_2 carrier gas with a flow rate of approximately 3 mL min^{-1}. CO was analysed using a HP-PLOT/Q column (0.53 mm diameter) attached to a HP-5 column (0.32 mm diameter). The GC oven temperature was kept constant at 45°C, He was used as carrier gas at an approximate flow rate of 2 mL min^{-1} and a thermal conductivity detector (TCD) was used. Methane (2% CH_4 in CO_2) was used as internal standard after calibration with different mixtures of known $CH_4/H_2/CO$ compositions.

External quantum yield (EQY) determination. Samples for photocatalytic formate decomposition were prepared in a quartz cuvette (A = 1 cm^2, 1 cm path length) according to the description below. The cuvette was sealed with a rubber septum and the solution was purged for 10 min in the dark

(N_2/2% CH_4 for H_2 evolution from FA solution or CO_2/2% CH_4 for CO evolution from aqueous solution). The cuvette was stirred at 600 RPM and irradiated with a Kodak projector lamp (CAROUSEL S-AV 2000, 250 W) equipped with a 460±10 nm narrow band pass filter (FB-460-10, Thorlabs); light intensity was measured with a power meter (ILT 1400, International Light Technologies). Product distribution was quantified through periodic headspace gas analysis (30 µL) by gas chromatography. The external quantum yield (EQY) was calculated according to equations 1 (for H_2) and 2 (for CO). Experimental controls confirmed both reactions to be light-dependent (Table S2, entries 15 and 16), however without detailed knowledge of the reaction mechanism for CO formation we are not aware of the number of photons required to form a CO molecule. We have therefore assumed that one photon is required for each CO molecule produced to avoid over-estimating the EQY_{CO}.

A) H_2 generation. An aqueous QD-MPA stock solution (139 µM = 26.0 mg mL^{-1}, 13.26 µL), was added to a quartz cuvette containing 2 mL of a solution of $NaHCO_2$ (4.0 M) and $CoCl_2 \cdot 6H_2O$ (0.5 mM) in formic acid and a magnetic stirrer bar.

B) CO generation. A QD-BF_4 stock solution in DMF (107 µM = 21.1 mg mL^{-1}, 17.15 µL) was added to a quartz cuvette containing a magnetic stirrer bar and the DMF was removed *in vacuo.* 2 mL of an aqueous solution of KOH (2.5 M) and $NaHCO_2$ (4.0 M) was added.

$$EQY_{H_2} = 100\% \times \frac{2 \times n\mathrm{H}_2 \times N_A \times h \times c}{t_{irr} \times \lambda \times I \times A} \qquad (1)$$

$$EQY_{CO} = 100\% \times \frac{n\mathrm{CO} \times N_A \times h \times c}{t_{irr} \times \lambda \times I \times A} \qquad (2)$$

Where nH_2 and nCO is the amount of H_2 and CO generated, respectively, N_A is Avogadro's constant, h is Planck's constant, c is the speed of light, t_{irr} is the irradiation time, λ is the wavelength, I is the light intensity and A is the irradiated area of the cuvette.

Infrared Spectrometric Analysis. FTIR spectra were recorded on a Thermo Scientific Nicolet iS50 FT-IR spectrometer. A stream of CO_2 (10 mL min^{-1}) was passed through a photoreactor containing 1 mL 1.0 M $NaH^{12}CO_2$ or $NaH^{13}CO_2$ in 2.5 M aqueous KOH and into a gas IR cell (SpecAc, 10 cm path length, equipped with KBr windows). The photoreactor was irradiated with a Kodak projector (250 W halogen lamp, I = 1 W cm^{-2}) equipped with a 420 nm cut-off filter (UQG Optics).

UV–Vis. UV–Vis spectra were recorded on a Varian Cary 50 UV–Vis spectrophotometer using quartz glass cuvettes.

TEM. Transmission Electron Microscopy (TEM) images were collected using a FEI Philips Tecnai 20 microscope, with 200 kV accelerating voltage. Samples were drop-cast onto holey carbon films (Agar Scientific).

XRD. X-ray diffraction (XRD) was conducted using an X'Pert PRO by PANalytical BV instrument.

XPS. X-ray photoelectron spectroscopy (XPS) was conducted by the National EPSRC XPS User's Service (NEXUS) at Newcastle University, UK, an EPSRC Mid-Range Facility. QD samples were loaded onto gold-coated silicon substrates and XPS analysis was performed using a K-Alpha (Thermo Scientific, East Grinstead, UK) spectrometer utilizing a monochromatic AlKα X-ray source (1486.6 eV, 400μm spot size, 36 W). Survey spectra were collected with a pass energy of 200 eV and 3 sweeps, while high resolution spectra were collected at a pass energy of 40 eV with 10 sweeps. Measurements were taken at 3 points on each sample surface to ensure consistency. Au(4f) from the substrate was used to calibrate the sample spectra with respect to binding energy. Software was used to create Gaussian fits to XPS data.

ICP. Inductively Coupled Plasma-Optical Emission Spectroscopy (ICP-OES) was carried out by Mr. Christopher Rolfe (Department of Geography, University of Cambridge) using a PerkinElmer Optima 2100TM DV spectrometer. Samples were digested in nitric acid for analysis.

Treatment of data. All analytical measurements were performed in triplicate and are given as mean ± standard deviation. A minimum σ of 10 % was assumed for all experiments even where triplicate experiments gave a σ of less than 10 %.

Supporting Figures

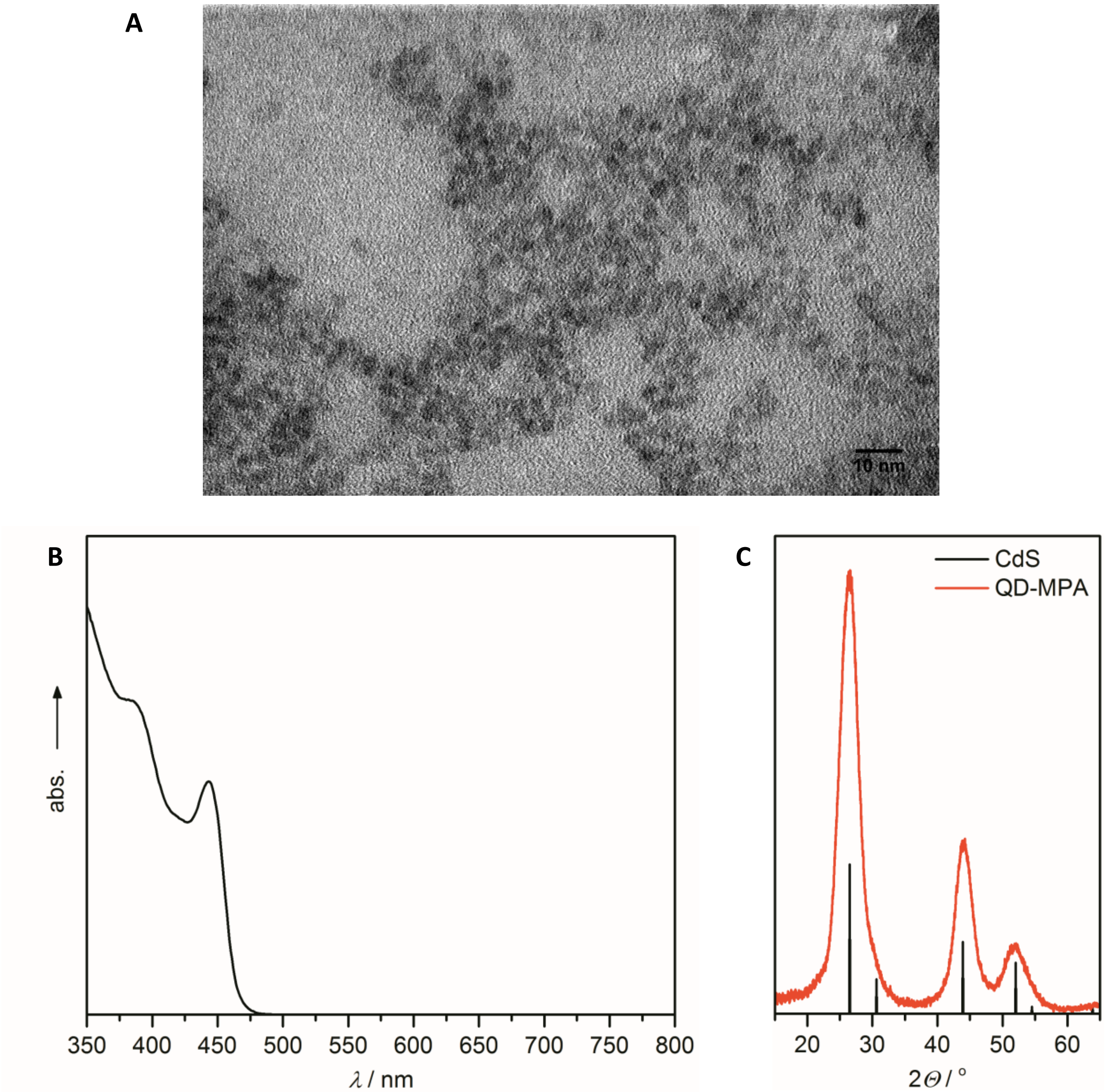

Figure S1. QD-MPA as prepared: A) TEM image; B) UV-vis spectrum in aqueous solution; C) powder XRD diffractogram.

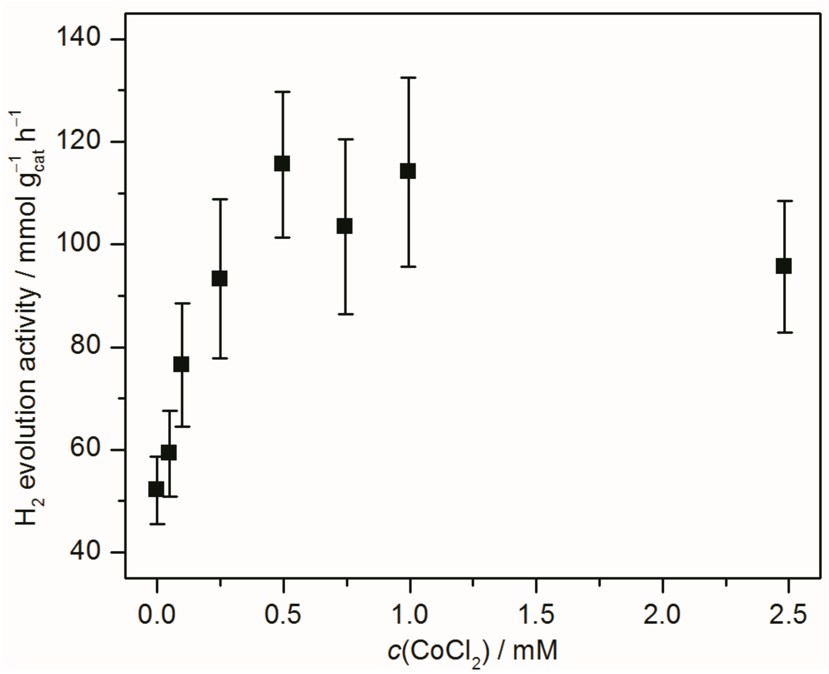

Figure S2. Photocatalytic H_2 generation using QD-MPA/$CoCl_2$ at varying co-catalyst loadings [100 mW cm^{-2} AM1.5G, λ>420 nm; 0.91 µM QD-MPA (171 µg mL^{-1}), 4.0 M $NaHCO_2$ in 2.0 mL formic acid; activity was determined after 1 h irradiation].

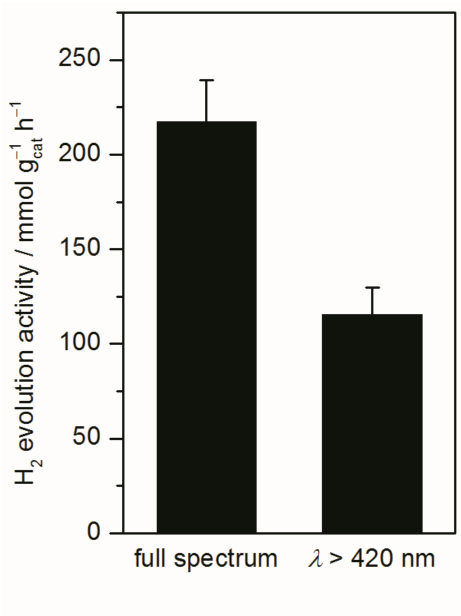

Figure S3. Photocatalytic H_2 generation using QD-MPA/$CoCl_2$ with or without a UV cutoff filter [100 mW cm^{-2} AM1.5G; 0.912 µM QD-MPA (176 µg mL^{-1}), 0.5 mM $CoCl_2{\cdot}6H_2O$, 4.0 M $NaHCO_2$ in 2.0 mL formic acid; activity was determined after 1 h irradiation].

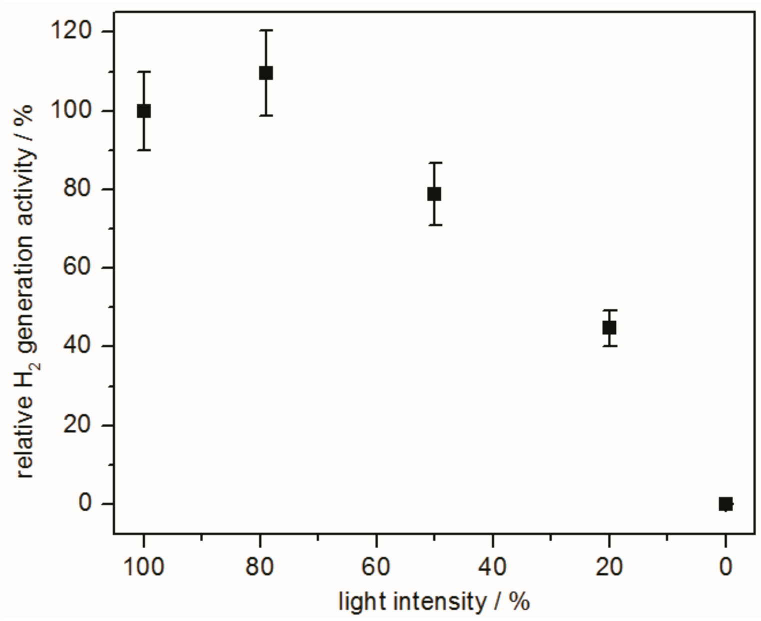

Figure S4. Photocatalytic H_2 generation using QD-MPA/$CoCl_2$ at varying light intensities [100% intensity ≈ 120 mW cm^{-2} AM1.5G, λ>420 nm, light intensity was varied using neutral density filters (UQG Optics); 0.912 µM QD-MPA (176 µg mL^{-1}), 0.5 mM $CoCl_2{\cdot}6H_2O$, 4.0 M $NaHCO_2$ in 2.0 mL formic acid. Activity was determined after 1 h irradiation and compared to a sample irradiated at 100% light intensity].

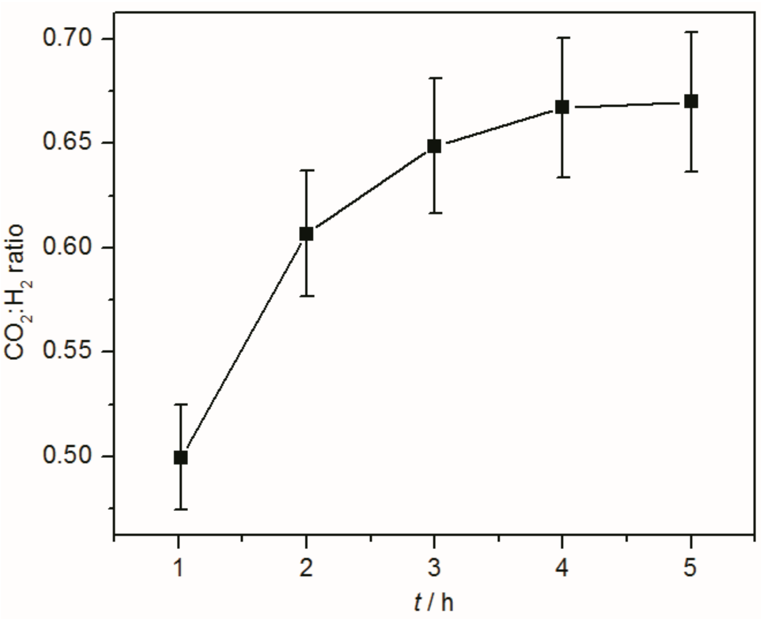

Figure S5. Relative CO_2 generation during photocatalytic H_2 generation using QD-MPA/$CoCl_2$ [100 mW cm^{-2} AM1.5G, λ>420 nm; 0.865 µM QD-MPA (162 µg mL^{-1}), 0.5 mM $CoCl_2{\cdot}6H_2O$, 4.0 M $NaHCO_2$ in 2.0 mL formic acid].

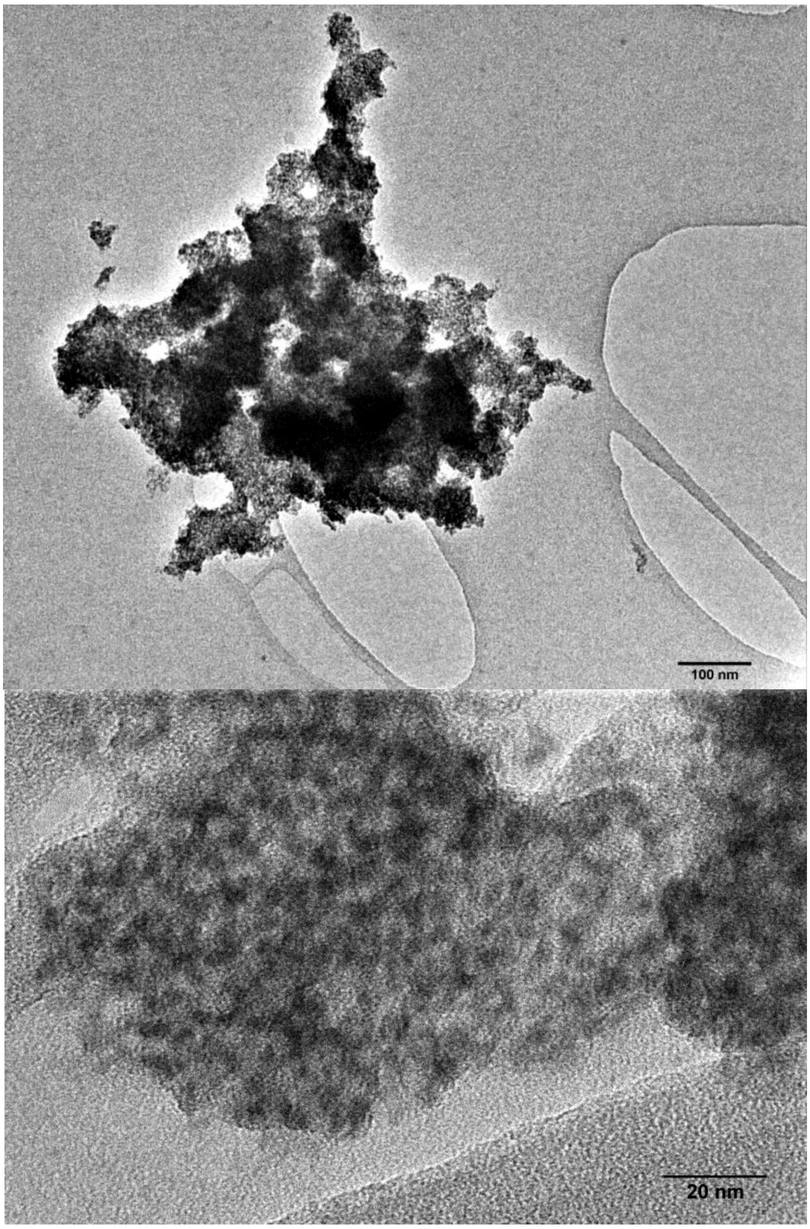

Figure S6. TEM images of QD-MPA/$CoCl_2$ after 4 h photocatalytic H_2 generation [100 mW cm^{-2} AM1.5G λ>420 nm; 1.50 μM QD-MPA (290 μg mL^{-1}), 0.5 mM $CoCl_2\cdot6H_2O$, 4.0 M $NaHCO_2$ in 2.0 mL formic acid.

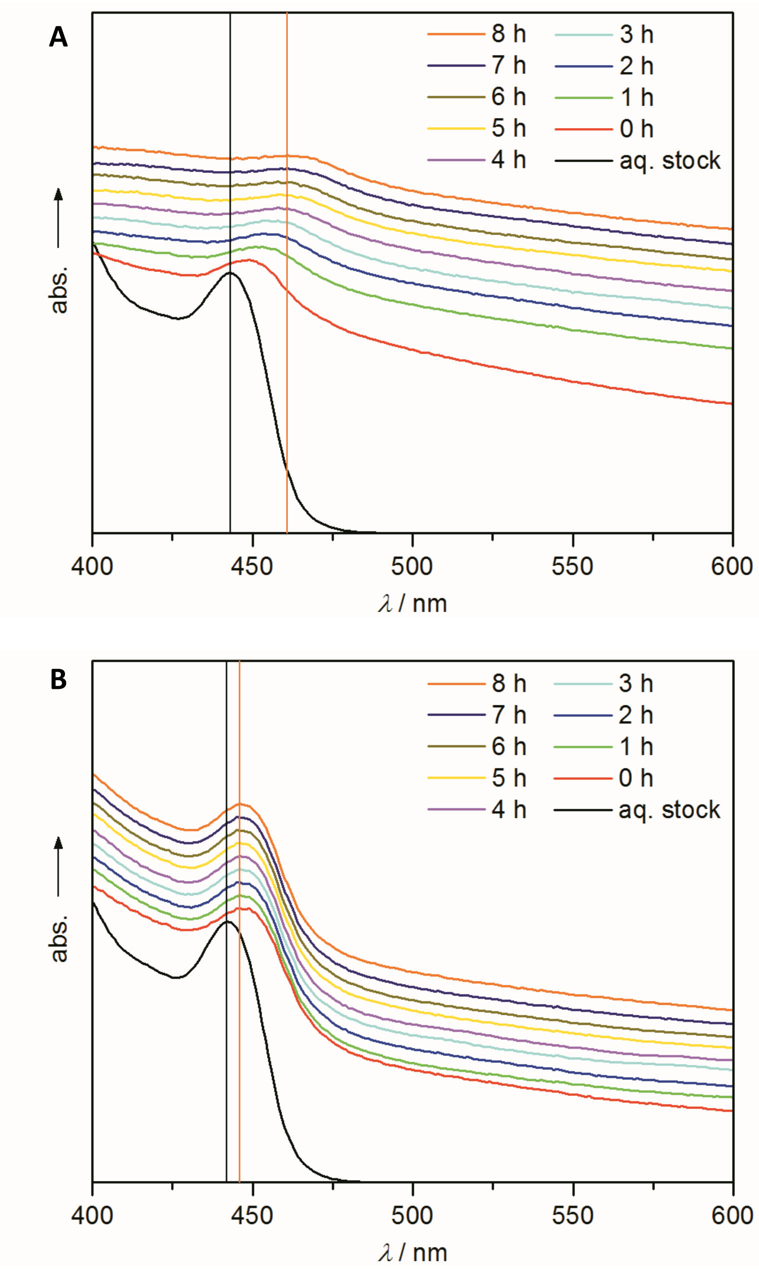

Figure S7. *In-situ* UV-vis spectra of QD-MPA/$CoCl_2$ during photocatalytic H_2 generation: A) without added MPA, B) in the presence of 140 mM MPA [100 mW cm^{-2} AM1.5G, λ>420 nm; 0.865 µM QD-MPA (162 µg mL^{-1}), 0.5 mM $CoCl_2 \cdot 6H_2O$, 4.0 M $NaHCO_2$ in 2.0 mL formic acid; absorbance was normalized with respect to the absorption maximum and spectra were stacked for clarity. For comparison, *ex-situ* spectra of as-prepared QD-MPA dispersed in pure water were included ("aq. stock"). Vertical lines represent absorption maxima of selected spectra].

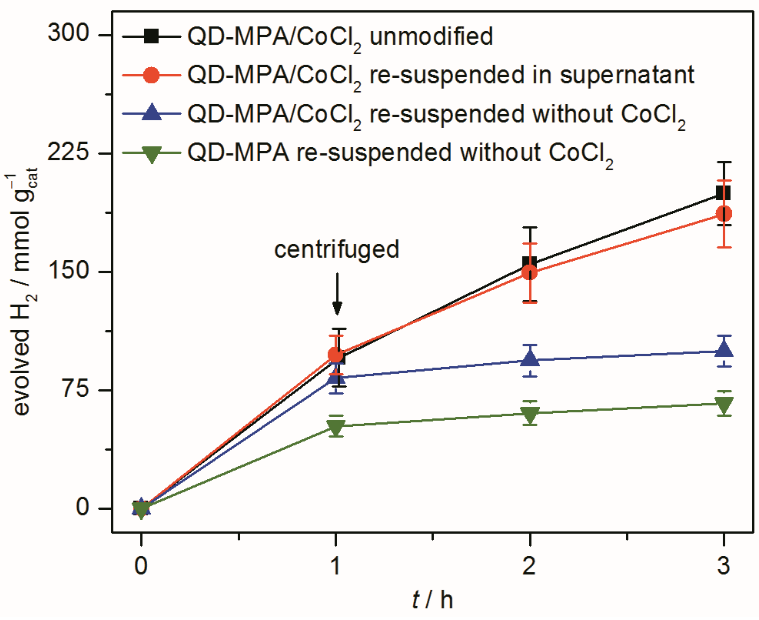

Figure S8. Separation experiments during the photocatalytic H_2 generation from formic acid/sodium formate using QD-MPA(/$CoCl_2$) [100 mW cm^{-2} AM1.5G; 0.865 μM QD-MPA (162 μg mL^{-1}), 0.5 mM $CoCl_2 \cdot 6H_2O$, 4.0 M $NaHCO_2$ in 2.0 mL formic acid].

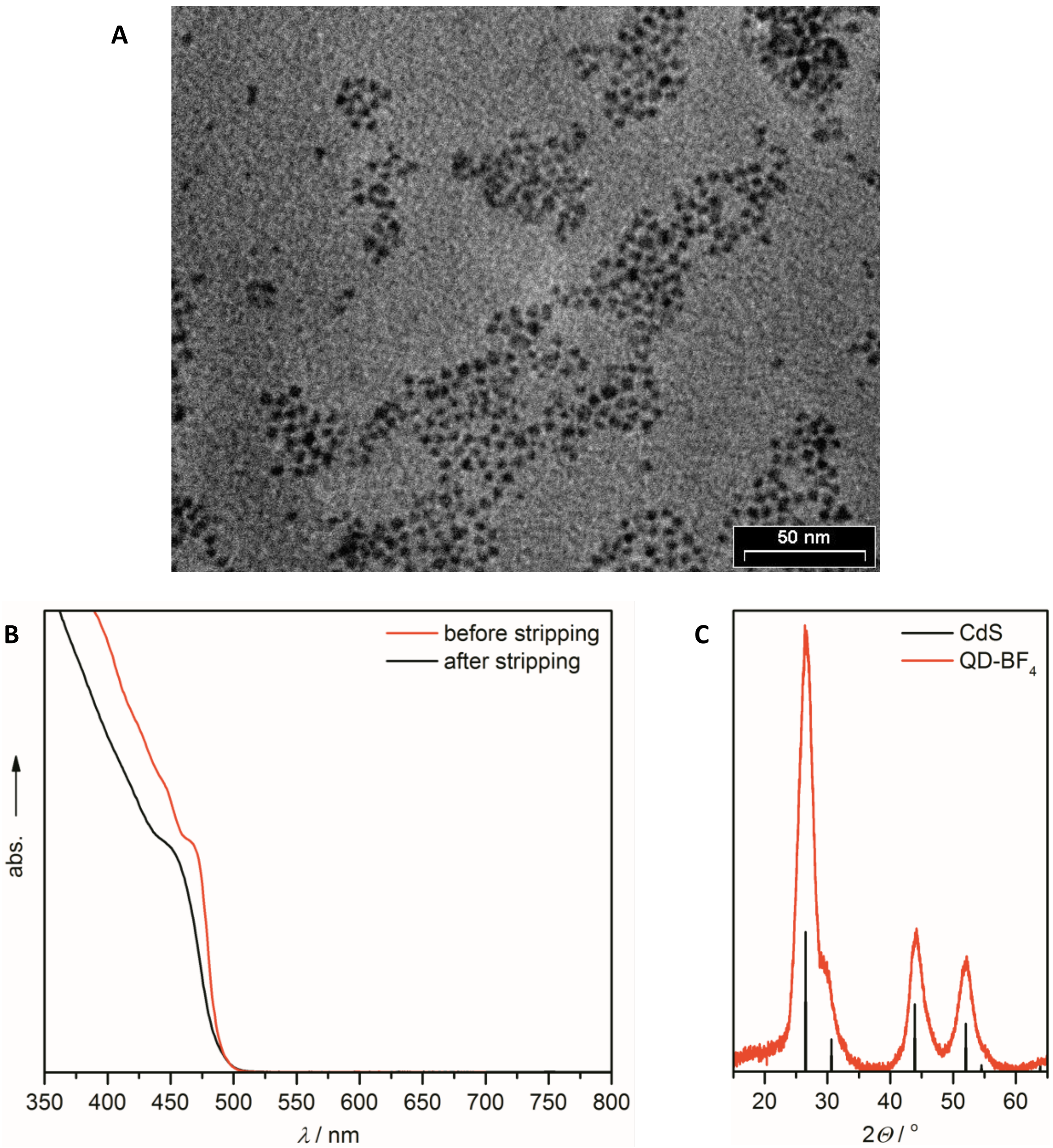

Figure S9. QD-BF_4 as prepared: A) TEM image; B) UV-vis spectrum before (in hexane solution) and after stripping (in DMF solution); C) powder XRD diffractogram.

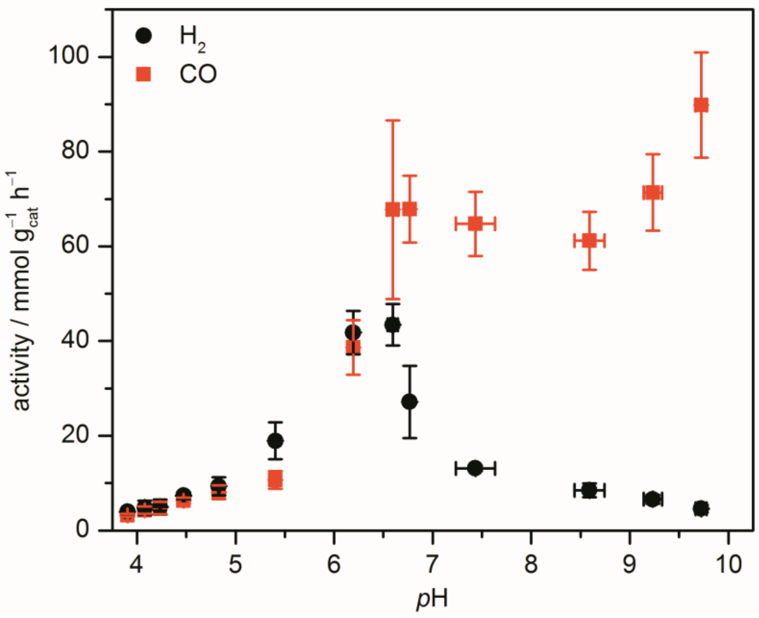

Figure S10. Photocatalytic decomposition of aqueous sodium formate using QD-BF_4 at varying pH [100 mW cm^{-2} AM1.5G, λ>420 nm; 0.61 µM QD-MPA (143 µg mL^{-1}) in 2.0 mL aqueous 4.0 M $NaHCO_2$, 2.5 M KOH/CO_2; pH was adjusted by adding conc. HCl before purging with CO_2 and measured after the purge. Activity was determined after 2 h irradiation].

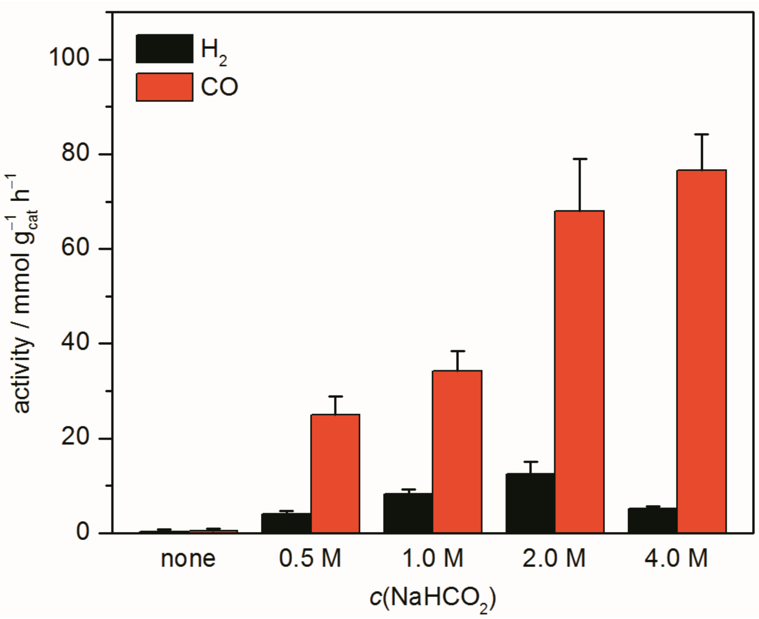

Figure S11. Photocatalytic decomposition of aqueous sodium formate using QD-BF_4 at varying formate concentrations [100 mW cm^{-2} AM1.5G, λ>420 nm; 0.67 µM QD-BF_4 (176 µg mL^{-1}) in 2.0 mL 2.5 M aqueous KOH/CO_2, pH not adjusted. Activity was determined after 2 h irradiation].

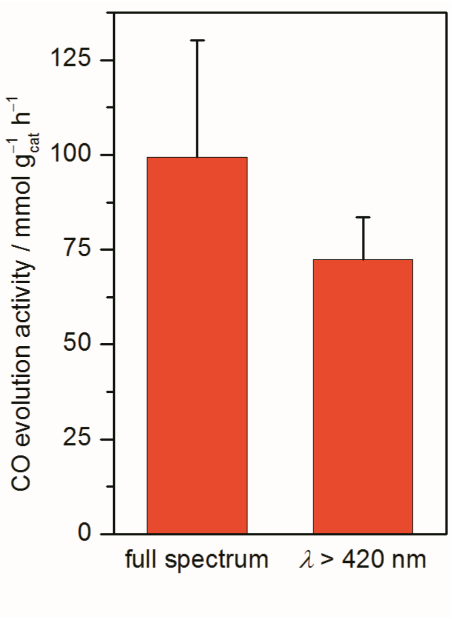

Figrue S12. Photocatalytic CO generation using QD-BF_4 with or without a UV cutoff filter [100 mW cm^{-2} AM1.5G; 0.611 µM QD-BF_4 (144 µg mL^{-1}), 4.0 M $NaHCO_2$ in 2.0 mL 2.5 M aqueous KOH/CO_2, pH 9.7. Activity was determined after 2 h irradiation].

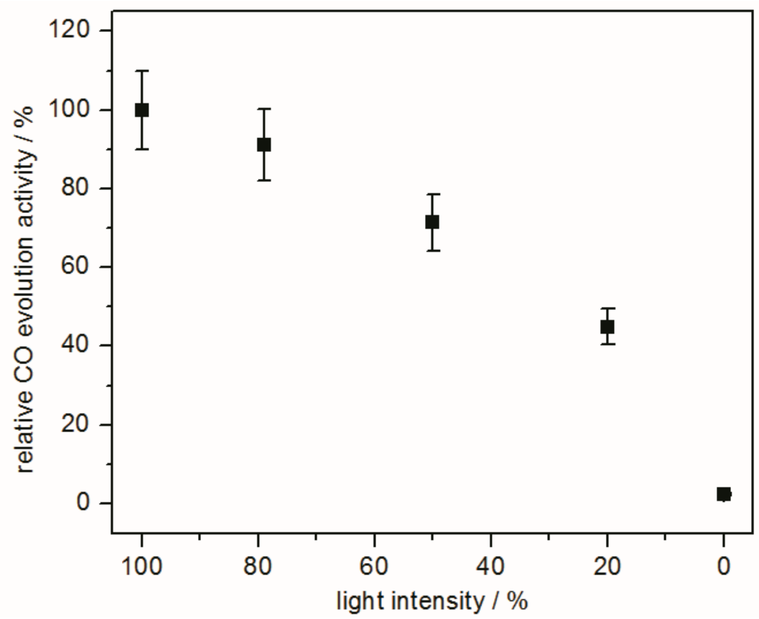

Figure S13. Photocatalytic CO generation using QD-BF_4 at varying light intensities [100% intensity ≈ 120 mW cm^{-2} AM1.5G, λ>420 nm, light intensity was varied using neutral density filters (UQG Optics); 0.918 µM QD-BF_4 (181 µg mL^{-1}), 4.0 M $NaHCO_2$ in 2.0 mL 2.5 M aqueous KOH/CO_2, pH 9.7. Activity was determined after 1 h irradiation and compared to a sample irradiated at 100% light intensity].

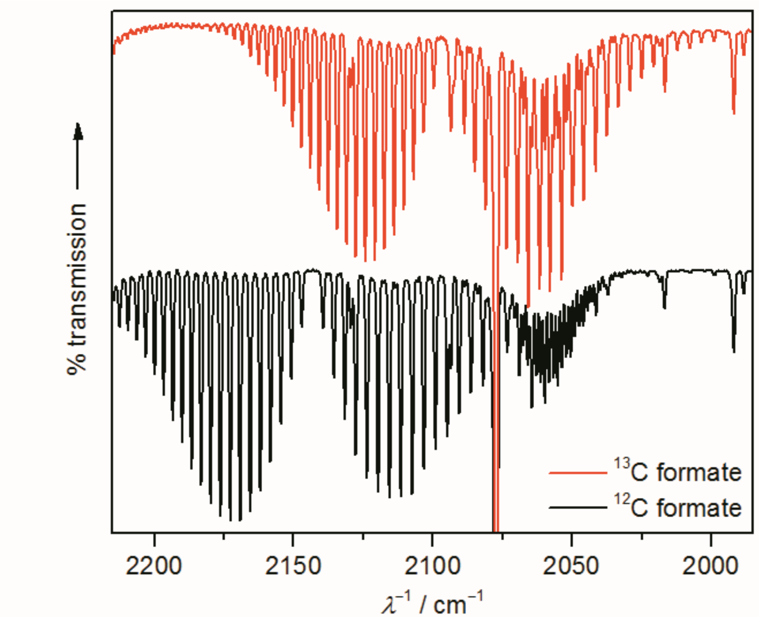

Figure S14. Gas-phase FT-IR spectra of photocatalytically generated CO using isotopically labelled sodium formate [halogen lamp, 1 W cm^{-2}, λ>420 nm; 1.10 µM QD-BF_4 (246 µg mL^{-1}), 0.5 M $NaH^{13}CO_2$ or $NaH^{12}CO_2$ in 1.0 mL 2.5 M aqueous KOH/CO_2, pH 9.7].

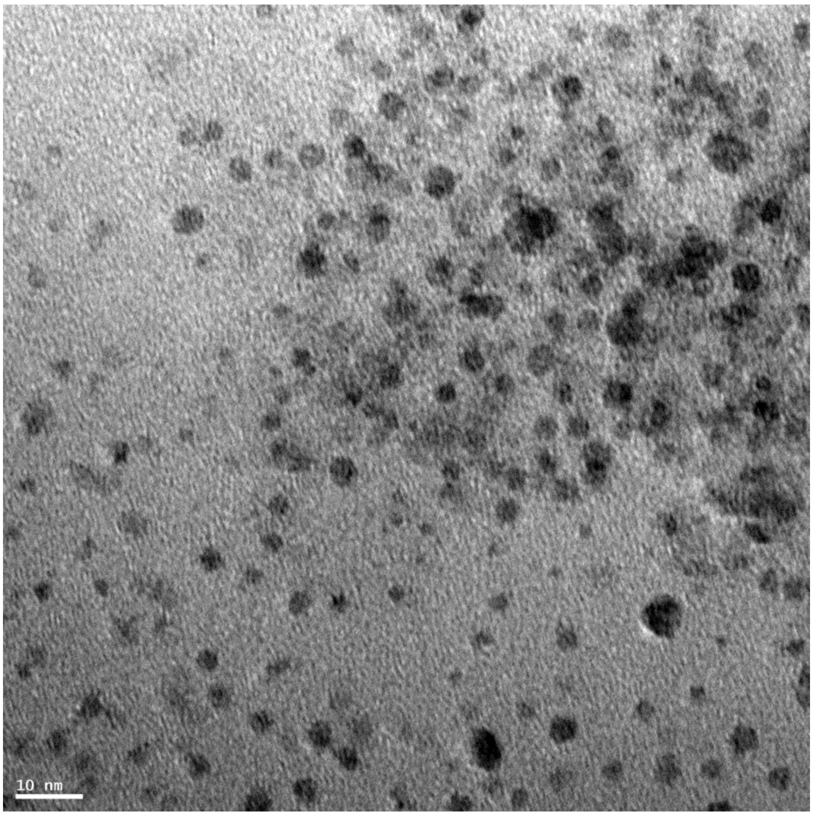

Figure S15. TEM image of QD-BF_4 after 6 h photocatalytic CO generation [100 mW cm^{-2} AM1.5G, λ>420 nm; 0.55 µM QD-BF_4 (123 µg mL^{-1}), 4.0 M $NaHCO_2$ in 2.0 mL 2.5 M aqueous KOH/CO_2, pH 9.7].

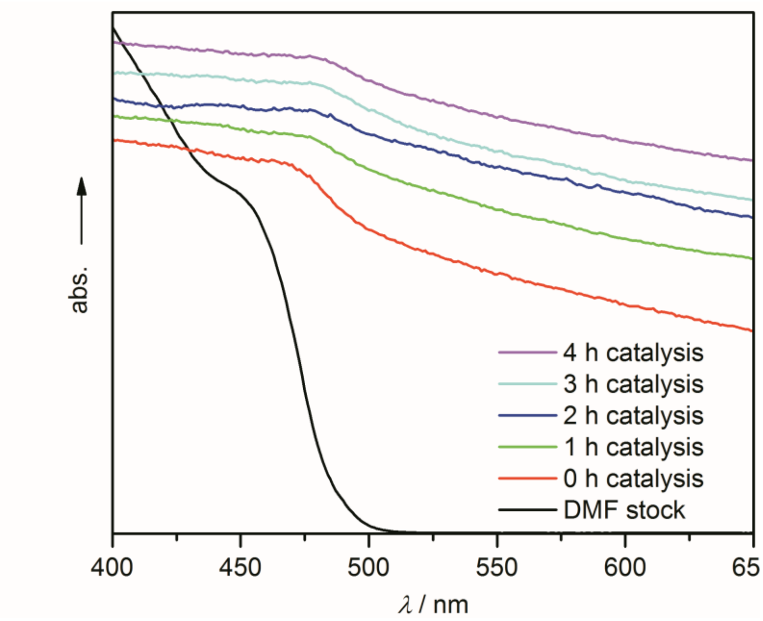

Figure S16. *In-situ* UV-vis spectra of QD-BF_4 during photocatalytic CO generation [100 mW cm^{-2} AM1.5G, λ>420 nm; 0.918 µM QD-BF_4 (181 µg mL^{-1}), 4.0 M $NaHCO_2$ in 2.0 mL 2.5 M aqueous KOH/CO_2, pH 9.7; absorbance was normalized with respect to the absorption maximum and spectra were stacked for clarity. For comparison, *ex-situ* spectrum of as-prepared QD-BF_4 dispersed in DMF was included ("DMF stock")].

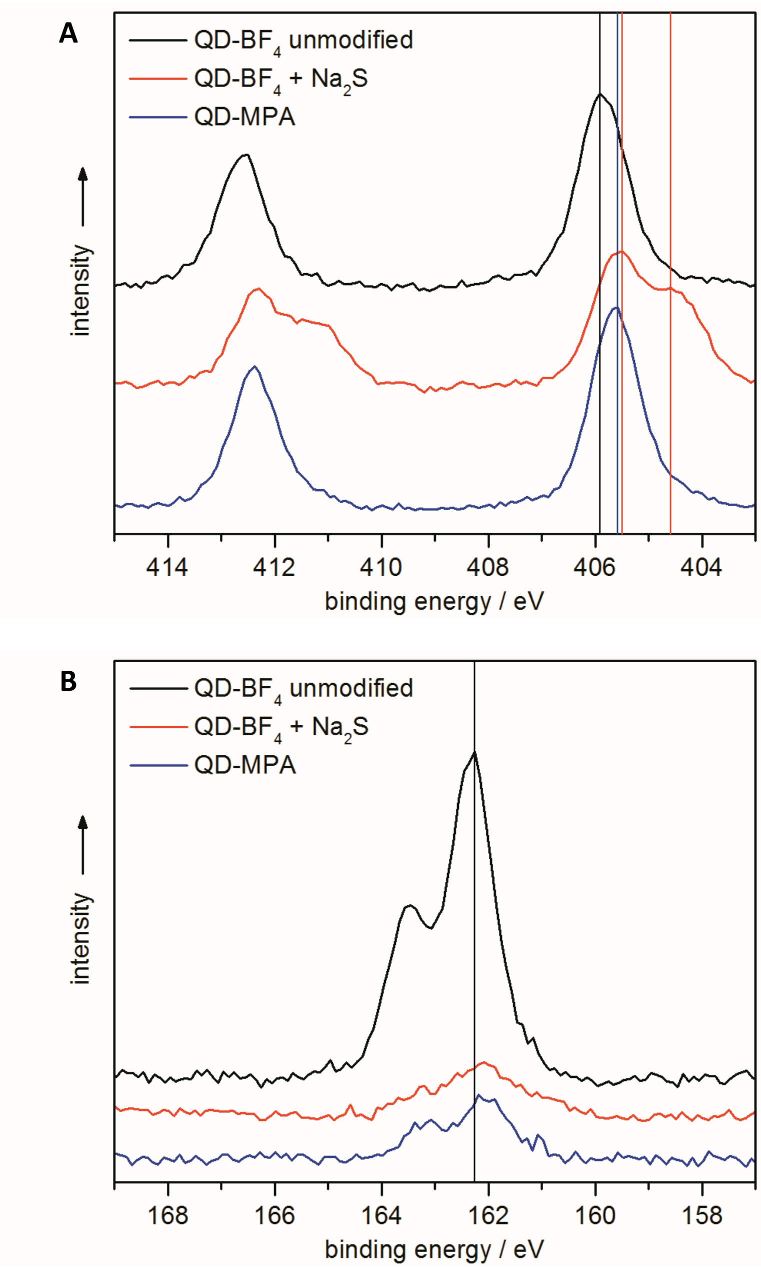

Figure S17. XPS spectra of unmodified QD-BF_4, Na_2S-poisoned QD-BF_4, and QD-MPA after 6 h photocatalysis; A) Cd(3d) region, B) S(2p) region [100 mW cm^{-2} AM1.5G, λ>420 nm; 0.55 µM QD-BF_4 (123 µg mL^{-1}) or 0.65 µM QD-MPA (126 µg mL^{-1}), 4.0 M $NaHCO_2$ in 2.0 mL 2.5 M aqueous KOH/CO_2, pH 9.7; vertical lines indicate selected peak positions].

Supporting Tables

Table S1. Photocatalytic H_2 generation from formic acid/sodium formate after 1 h of irradiation using different co-catalyst concentrations [100 mW cm^{-2} AM1.5G, λ>420 nm; 4.0 M $NaHCO_2$, 0.91 µM QD-MPA (171.3 µg mL^{-1}) in 2.0 mL formic acid].

entry	$c(CoCl_2 \cdot 6H_2O)$ / mM	$nH_2 \pm \sigma$ / µmol	H_2 activity $\pm \sigma$[a] / mmol H_2 $g_{catalyst}^{-1}$ h^{-1}	$TON_{QD} \pm \sigma$[b] / mol H_2 mol_{QD}^{-1}
1[b]	0	17.0±2.1	52.1±6.6	9,760±1,230
2	0.05	20.9±3.0	59.2±8.4	8,370±1,620
3	0.10	27.0±4.2	76.5±12.0	14,700±2,300
4	0.25	33.0±5.5	93.3±15.5	17,900±3,000
5	0.50	40.8±5.0	116±14	22,200±2,700
6	0.75	36.6±6.0	104±17	19,900±3,300
7	1.0	40.4±6.5	114±19	22,000±3,600
8	2.5	34.0±4.5	95.7±12.7	18,500±2,500

[a] Calculated using the mass of QD-MPA + $CoCl_2 \cdot 6H_2O$; [b] 0.87 µM QD-MPA (166 µg CdS mL^{-1}); [b] $TON_{QD} = nH_2 / n$QD-MPA.

Table S2. Photocatalytic H_2 and CO generation using different solvents and photocatalysts after 1 h irradiation (100 mW cm^{-2} AM1.5G, in 2.0 mL solvent).

entry	photocatalyst, co-catalyst	solvent[a]	light	H_2 activity[b] ± σ / mmol H_2 $g_{catalyst}^{-1}$ h^{-1}	CO activity[b] ± σ / mmol CO $g_{catalyst}^{-1}$ h^{-1}
QD-MPA					
1	QD-MPA, 162 µg mL^{-1} no co-catalyst	SF/FA	λ>420 nm	52.1±6.6	0.61±0.13
2	QD-MPA, 162 µg mL^{-1} 0.5 mM $CoCl_2 \cdot 6H_2O$	SF/FA	λ>420 nm	94.0±17.9	0.59±0.14
3	QD-MPA, 176 µg mL^{-1} 0.5 mM $CoCl_2 \cdot 6H_2O$	SF/FA	full spectrum	218±22	2.45±0.25
4	QD-MPA, 162 µg mL^{-1} 0.5 mM $CoCl_2 \cdot 6H_2O$	neat FA	λ>420 nm	1.41±0.31[b]	0.050±0.005[c]
5	QD-MPA, 90.7 µg mL^{-1} no co-catalyst	SF/H_2O pH 14[d]	λ>420 nm	0.066±0.036	0.088±0.026
6	QD-MPA, 162 µg mL^{-1} 0.5 mM $CoCl_2 \cdot 6H_2O$	SF/H_2O pH 14[d]	λ>420 nm	0.016±0.002	0.022±0.004
QD-BF_4					
7	QD-BF_4, 61.1 µg mL^{-1} no co-catalyst	SF/FA	λ>420 nm	11.6±1.6	1.18±0.14
8	QD-BF_4, 61.1 µg mL^{-1} 0.25 mM $CoCl_2 \cdot 6H_2O$	SF/FA	λ>420 nm	80.5±12.8	1.60±0.32
9	QD-BF_4, 12.3 µg mL^{-1} no co-catalyst	SF/H_2O pH 9.7	λ>420 nm	3.93±0.44	102±13
10	QD-BF_4, 12.3 µg mL^{-1} 0.5 mM $CoCl_2 \cdot 6H_2O$	SF/H_2O pH 9.7	λ>420 nm	2.67±1.48	12.8±3.1
controls					
11	bulk CdS, 26.9 µg mL^{-1} 0.5 mM $CoCl_2 \cdot 6H_2O$	SF/FA	λ>420 nm	0.229±0.067	not determined
12	bulk CdS, 13.9 µg mL^{-1} no co-catalyst	SF/H_2O pH 9.7	λ>420 nm	0.173±0.017	3.52±0.63
13	none 0.5 mM $CoCl_2 \cdot 6H_2O$	SF/FA	λ>420 nm	no H_2 detected	no CO detected
14	none	SF/H_2O pH 9.7	λ>420 nm	no H_2 detected	no CO detected
15	QD-MPA, 167 µg mL^{-1} 0.5 mM $CoCl_2 \cdot 6H_2O$	SF/FA	dark	<0.01[e]	no CO detected
16	QD-BF_4, 10.9 µg mL^{-1} no co-catalyst	SF/H_2O pH 9.7	dark	no H_2 detected	<0.1[e]

[a] SF/FA = 4.0 M $NaHCO_2$ in formic acid, FA = formic acid, SF/H_2O = 4.0 M aqueous $NaHCO_2$ pH 14, 2.5 M KOH; SF/H_2O pH 9.7= 4.0 M aqueous $NaHCO_2$, 2.5 M KOH/CO_2 pH 9.7;[b] calculated using the mass of QD + $CoCl_2 \cdot 6H_2O$; [c] measured after 2 hours; [d] only traces of products were observed at pH 9.7; [e] limit of detection under these conditions.

Table S3. External quantum yield (EQY) determination for the photocatalytic H_2 generation from formic acid/sodium formate using QD-MPA/$CoCl_2$ [I=3.21±0.20 mW cm^{-2}, λ=460±10 nm, A=1 cm^2; 0.91 µM QD-MPA (176 µg mL^{-1}), 0.5 mM $CoCl_2·6H_2O$ 4.0 M $NaHCO_2$ in 2.0 mL formic acid. Data is given as mean ± s.d. of two independent measurements].

time / h	$nH_2 \pm \sigma$ / µmol	EQY_{H2} / %
1	2.37±0.45	10.2±1.8
2	4.42±0.41	19.0±1.9
3	4.83±0.25	20.8±2.1
4	5.09±1.00	21.9±4.0
5	5.36±0.32	23.1±2.3
average for 2-5 h	4.93±0.67	21.2±2.7

Table S4. Cumulative long-term photocatalytic H_2 generation from formic acid/sodium formate using QD-MPA (100 mW cm^{-2} AM1.5G, λ>420 nm; 4.0 M $NaHCO_2$ in 2.0 mL formic acid; samples were re-purged with N_2 (2% CH_4) after each measurement).

time / h	evolved $H_2 \pm \sigma$[a] / mol H_2 $g_{catalyst}^{-1}$	evolved CO $\pm \sigma$[a] / mol CO $g_{catalyst}^{-1}$	$TON_{QD} \pm \sigma$[b] / mol H_2 mol_{QD}^{-1}
	0.91 µM QD-MPA (176 µg mL^{-1}), no co-catalyst		
24	0.114±0.011	0.00471±0.00047	22,000±2,000
48	0.147±0.015	0.00579±0.00058	28,200±2,800
72	0.167±0.017	0.00657±0.00066	32,100±3,200
96	0.181±0.018	0.00707±0.00071	34,700±3,500
132	0.201±0.20	0.00824±0.00082	38,700±3,900
168	0.216±0.022	0.00927±0.00093	41,500±4,200
	0.91 µM QD-MPA (176 µg mL^{-1}), 0.5 mM $CoCl_2 \cdot 6H_2O$		
24	0.808±0.099	0.0134±0.0026	156,000±19,000
48	1.05±0.11	0.0168±0.0023	202,000±20,000
72	1.22±0.12	0.0197±0.0021	235,000±24,000
96	1.34±0.13	0.0220±0.0026	258,000±26,000
132	1.49±0.15	0.0262±0.0026	286,000±29,000
168	1.63±0.16	0.0319±0.0032	314,000±31,000
	0.90 µM QD-MPA (173 µg mL^{-1}), 0.5 mM $CoCl_2 \cdot 6H_2O$, 140 mM MPA		
24	0.413±0.064	0.0147±0.0015	79,400±12,000
48	0.785±0.078	0.0260±0.0026	151,000±15,000
72	1.22±0.12	0.0324±0.0032	236.000±24,000
96	1.69±0.17	0.0370±0.0037	326,000±33,000
132	2.48±0.41	0.0466±0.0047	477,000±78,000
168	3.14±0.49	0.0566±0.0057	605,000±95,000

[a] Calculated on the basis of the mass of QD-MPA + $CoCl_2 \cdot 6H_2O$; [b] $TON_{QD} = nH_2 / n$QD-MPA.

Table S5. Comparison of selected literature photocatalysts for FA-to-H_2 conversion at room temperature.

catalyst	H_2 production[a] [mmol H_2 $g_{catalyst}^{-1}$ h^{-1}]	selectivity[b] [%]	EQY [%]	light	lifetime [h]	ref
Pd@C_3N_4	53.4	100	n/a	>400 nm	6	[5]
Pd@Au-NRs	10	100	n/a	>460 nm, 1 sun	n/a	[6]
AuPd-TiO_2 NW	17.7	99.7	15.6 (365 nm)	AM1.5, 1 sun	9	[7]
Au-TiO_2 NW	3.9	90.7	8.1 (365 nm)	AM1.5, 1 sun	9	[7]
Pd-TiO_2 NW	10.9	98.2	11.6 (365 nm)	AM1.5, 1 sun	9	[7]
TiO_2 NW	0.80	69.6	0.35 (365 nm)	AM1.5, 1 sun	9	[7]
Pd-Si	1.1	100	n/a	visible	5	[8]
Pt-Si	0.001	n/a	0.02 (633 nm)	>390 nm, 2 sun	100	[9]
Pt-TiO_2	1.62	n/a	n/a	UV	5	[10]
Pt-$(CuIn)_{0.2}Zn_{1.6}S_2$	0.72	n/a	n/a	>420 nm	10	[11]
Rh-N:TiO_2	0.746	98	n/a	230-440 nm	4	[12]
Cu-TiO_2	0.83	n/a	n/a	UV	5	[13]
$RuCl_2(C_6H_6)]_2$ + 12 PPh_3	153.9	n/a	n/a	>380	5	[14]
$HCo[(PPh(OEt)_2]_4$	0.59	n/a	n/a	>275 nm	6	[15]
Co_3O_4-$LiNbO_3$	0.78	n/a	n/a	200-600 nm	2.5	[16]
$Fe_3(CO)_{12}$/PPh_3/tpy	2.7	"trace CO"	n/a	>385 nm	24	[17]
Bulk CdS	0.078	n/a	n/a	>400 nm	12	[18]
CdS-TNT TiO_2	0.56	n/a	5.1	>430 nm	8	[19]
Pt-CdS	1.84	n/a	20 (470 nm)	>320 nm	10	[20]
Pt-CdS	0.85	83	n/a	>400 nm	20	[21]
Pt-CdS NR	4.46	n/a	13.9 (400-700 nm)	>420 nm	50	[22]
CdS-NR	0.22	n/a	n/a	>420 nm	n/a	[22]
Pt-CdS-QD	1.22	n/a	21.4 (420 nm)	>420 nm	30	[23]
Pt-CdS@Al-MHS	0.31	n/a	2.0 (420 nm)	>420 nm	6	[24]
Ru-CdS@Al-HMS	0.541	n/a	5.92 (420 nm)	>420 nm	6	[25]
CdS@Al-HMS	0.13	n/a	n/a	>420 nm	6	[25]
Ru-CdS/ZnS NP	5.85±0.09	n/a	20	>420 nm	40	[26]
CdS/ZnS NP	1.24±0.02	n/a	n/a	>420 nm	40	[26]
CdS-TNT + WO_3	0.619	n/a	n/a	>420 nm	3	[27]
Pt-CdS-TNT	4.26	n/a	n/a	>420 nm	3	[27]
hydrogenase-CdS	0.356	20	3.1 (IQE)	400-600 nm	3.5	[28]
CdS	0.036	3.2	1.9 (IQE)	400-600 nm	3.5	[28]
QD-MPA	52.1±6.6	98.8±0.1	n/a	AM1.5G >420 nm	>168	This work
QD-MPA/$CoCl_2$	116±14	99.4±0.1	21.2±2.7 (460 nm)	AM1.5G >420 nm	>168	This work
QD-MPA/$CoCl_2$	218±22	98.9±0.1	n/a	AM1.5G	>24	This work

[a] For an accurate comparison, published data was converted to gravimetric activity using the mass of the entire photocatalyst used for reaction; [b] selectivity = 100% * nH_2 / (nH_2 + nCO).

Table S6. Separation experiments during the photocatalytic H_2 generation from formic acid/sodium formate using QD-MPA [100 mW cm^{-2} AM1.5G, λ>420 nm; 162 µg mL^{-1} QD-MPA (0.865 µM), with or without 0.5 mM $CoCl_2 \cdot 6H_2O$, 4.0 M $NaHCO_2$ in 2.0 mL formic acid].

time / h	QD-MPA/$CoCl_2$ H$_2$ activity ± σ[a] / mmol H_2 $g_{catalyst}^{-1}$ h^{-1}	QD-MPA/$CoCl_2$ H$_2$ activity ± σ[a] / mmol H_2 $g_{catalyst}^{-1}$ h^{-1}	QD-MPA/$CoCl_2$ H$_2$ activity ± σ[a] / mmol H_2 $g_{catalyst}^{-1}$ h^{-1}	QD-MPA/$CoCl_2$ H$_2$ activity ± σ[a] / mmol H_2 $g_{catalyst}^{-1}$ h^{-1}	QD-MPA H$_2$ activity ± σ / mmol H_2 $g_{catalyst}^{-1}$ h^{-1}
1	95.6±18.3	97.3±12.4	82.7±9.5	115±21	52.1±6.6
	unmodified	centrifuged, solid recombined with same supernatant.[b]	centrifuged, solid re-dispersed in fresh FA/sodium formate without co-catalyst	centrifuged, continued with filtered supernatant only	centrifuged, solid re-dispersed in fresh FA/sodium formate without co-catalyst
2	59.3±11.1	52.1±8.2	5.49±0.55	0.0001±0.0001	4.22±0.48
3	44.8±10.5	37.3±0.4	2.01±0.20	0.006±0.008	2.07±0.21

[a] Calculated on the basis of the mass of QD-MPA + $CoCl_2 \cdot 6H_2O$ and the individual amount of H_2 evolved in the respective hour; [b] As a control to account for potential losses during transfer to and from the centrifugation vial, the solid was re-dispersed in the same supernatant.

Table S7. Photocatalytic CO generation from aqueous sodium formate after 2 h of irradiation at different pH [100 mW cm^{-2} AM1.5G, λ>420 nm; 0.61 µM QD-BF_4 (143 µg mL^{-1}) in 2.0 mL aqueous 4.0 M $NaHCO_2$, 2.5 M KOH/CO_2, pH adjusted with conc. HCl before purging with CO_2 and measured after the purge].

entry	pH[a]	CO activity ± σ / mmol CO $g_{catalyst}^{-1}$ h^{-1}	H$_2$ activity ± σ / mmol H_2 $g_{catalyst}^{-1}$ h^{-1}	TON_{QD} ± σ[b] / mol CO mol_{QD}^{-1}
1	9.72±0.06	89.8±11.1	4.63±0.81	42,000±5,200
2[c]	9.72±0.06	99.4±30.8	7.34±1.79	46,500±14,400
3	9.23±0.09	71.4±8.1	6.59±0.90	33,400±3,800
4	8.59±0.15	61.2±6.1	8.53±1.47	28,600±2,900
5	7.43±0.20	64.7±6.7	13.1±1.31	30,300±3,200
6	6.77±0.01	67.93±7.1	27.1±7.7	37,800±3,300
7	6.60±0.05	67.7±18.9	43.4±4.34	31,700±8,800
8	6.19±0.01	38.6±5.8	41.8±4.5	18,100±2,700
9	5.40±0.03	10.7±1.8	19.0±3.87	5,000±860
10	4.83±0.01	8.09±1.44	9.36±1.88	3,790±670
11	4.47±0.01	6.29±0.63	7.39±0.74	2,950±290
12	4.23±0.01	4.77±1.31	4.98±1.57	2,230±610
13	4.08±0.01	4.31±0.78	4.84±1.50	2,020±370
14	3.90±0.01	3.24±0.32	3.98±0.47	1,520±150

[a] pH after 10 min purge with CO_2 (2% CH_4); [b] TON_{QD} = nCO / nQD-BF_4; [c] full solar spectrum used.

Table S8. Photocatalytic CO generation from aqueous sodium formate after 2 h of irradiation at different formate concentrations [100 mW cm^{-2} AM1.5G, λ>420 nm; 0.67 μM QD-BF_4 (176 μg mL^{-1}) in 2.0 mL aqueous 4.0 M $NaHCO_2$, 2.5 M KOH/CO_2, pH not adjusted].

entry	c($NaHCO_2$) / M	CO activity ± σ / mmol CO $g_{catalyst}^{-1}$ h^{-1}	H_2 activity ± σ / mmol H_2 $g_{catalyst}^{-1}$ h^{-1}	TON_{QD} ± σ[a] / mol CO mol_{QD}^{-1}
1[b]	~0.0[c]	0.532±0.382	0.318±0.449	118±85
2	0.5	24.9±4.0	4.07±0.62	13,100±2,100
3	1.0	34.2±4.2	8.33±0.83	18,000±2,200
4	2.0	67.9±11.1	12.5±2.5	35,700±5,800
5	4.0	76.5±7.6	5.17±0.52	40,200±3,000

[a] TON_{QD} = *n*CO / *n*QD-BF_4; [b] 0.055 μM QD-BF_4 (12.3 μg mL^{-1}); [c] Small amounts of formate are present in solution due to residual N,N-dimethylformamide solvent on the particle surface.

Table S9. Long-term photocatalytic CO generation from sodium formate using QD-BF_4 [100 mW cm^{-2} AM1.5G, λ>420 nm; 0.0611 μM QD-BF_4 (14.4 μg mL^{-1}), in 2.0 mL aqueous 4.0 M $NaHCO_2$, 2.5 M KOH/CO_2, pH 9.7].

time / h	evolved CO ± σ / mol CO $g_{catalyst}^{-1}$	evolved H_2 ± σ / mol H_2 $g_{catalyst}^{-1}$	TON_{QD} ± σ[a} / mol CO mol_{QD}^{-1}
20	1.78±0.18	0.190±0.019	417,000±42,000
48	4.09±0.42	0.468±0.054	957,000±99,000
80	6.89±0.78	0.776±0.079	1,610,000±180,000
104	9.00±0.90	1.00±0.10	2,110,000±210,000
142	11.9±1.2	1.37±0.14	2,790,000±280,000
166	14.0±1.4	1.69±0.17	3,270,000±330,000

[a] TON_{QD} = *n*CO / *n*QD-BF_4.

Table S10. External quantum yield (EQY) determination for the photocatalytic CO generation from sodium formate using QD-BF_4 [I=3.43 mW cm^{-2}, λ=460±10 nm, A=1 cm^2; 0.912 µM QD-BF_4 (180 µg mL^{-1}) in 2.0 mL aqueous 4.0 M $NaHCO_2$, 2.5 M KOH/CO_2, pH 9.7. Data is given as mean ± s.d. of two independent measurements].

time / h	nCO ± σ / µmol	nH_2 ± σ / µmol	EQY_{CO} / %	EQY_{H2} / %
1	7.16±0.72	0.224±0.037	17.2±1.7	1.08±0.18
2	7.57±0.76	0.689±0.0176	18.2±1.8	2.24±0.67
3	8.36±0.84	1.20±0.33	20.1±2.0	2.46±0.75
4	7.53±0.75	1.88±0.67	18.1±1.8	3.28±1.65
5	9.42±1.87	2.37±0.24	22.7±4.5	2.34±0.30
6	8.12±0.81	2.87±0.53	19.6±2.0	2.41±0.43
average for 2-6h	8.20±1.13	1.80±0.99	19.7±2.7	2.55±0.97

Table S11. *In-situ* inhibition of the photocatalytic CO generation from sodium formate using QD-BF_4 [100 mW cm^{-2} AM1.5G, λ>420 nm; 0.055 µM QD-BF_4 (12.3 µg mL^{-1}) in 2.0 mL aqueous 4.0 M $NaHCO_2$, 2.5 M KOH/CO_2, pH 9.7].

time / h	evolved CO ± σ / mmol CO $g_{catalyst}^{-1}$	evolved H_2 ± σ / mmol H_2 $g_{catalyst}^{-1}$	evolved CO ± σ / mmol CO $g_{catalyst}^{-1}$	evolved H_2 ± σ / mmol H_2 $g_{catalyst}^{-1}$
1	90.2±9.0	3.95±0.62	82.2±11.1	3.47±0.35
	undisturbed		111 mM Na_2S added	
2	186±4.9	6.81±1.64	90.2±11.9	3.96±0.40
3	317±32	9.81±2.36	88.4±14.6	4.04±0.46
4	435±80	13.0±4.0	94.6±15.6	4.62±0.46

Table S12. Photocatalytic CO generation from sodium formate using QD-BF_4 in the presence of different inhibitors [100 mW cm^{-2} AM1.5G, λ>420 nm; 0.055 µM QD-BF_4 (12.3 µg mL^{-1}) in 2.0 mL aqueous 4.0 M $NaHCO_2$, 2.5 M KOH/CO_2, pH 9.7].

entry	inhibitor	CO activity ± σ / mmol H_2 $g_{catalyst}^{-1}$ h^{-1}	H_2 activity ± σ / mmol H_2 $g_{catalyst}^{-1}$ h^{-1}
1	none	102±13	3.93±0.44
2	111 mM Na_2S	13.2±4.9	0.533±0.181
3	111 mM MPA	16.5±6.48	2.02±0.39
4	83.3 mM Na_2EDTA	16.8±2.1	1.30±0.44

Table S13. Quantitative analysis of binding energy and Cd/S content of QD-BF_4 before and after Na_2S poisoning and QD-MPA from XPS spectroscopy after 6 h of photocatalytic CO generation. [100 mW cm^{-2} AM1.5G, λ>420 nm; 0.55 µM QD-BF_4 (123 µg mL^{-1}) or 0.65 µM QD-MPA (126 µg mL^{-1}), 4.0 M $NaHCO_2$ in 2.0 mL 2.5 M aqueous KOH/CO_2, pH 9.7; peak positions determined by fitting Gaussian peak functions].

sample		binding energy / eV		Cd:S ± σ / relative atom %
	Cd($3d_{3/2}$) ± σ	Cd($3d_{5/2}$) ± σ	S($2p_{3/2}$) ± σ	
QD-BF_4 unmodified	412.621±0.013	405.868±0.014	162.300±0.008	50.3:49.7±0.5
QD-BF_4 + Na_2S	412.371±0.022	405.628±0.015	162.019±0.084	47:53±8
QD-MPA	412.328±0.103	405.610±0.045	161.888±0.182	49:51±5

Supporting References

[1] L. Huang, X. Wang, J. Yang, G. Liu, J. Han, C. Li, *J. Phys. Chem. C* **2013**, *117*, 11584-11591.
[2] J. Aldana, N. Lavelle, Y. Wang, X. Peng, *J. Am. Chem. Soc.* **2005**, *127*, 2496-2504.
[3] W. W. Yu, L. Qu, W. Guo, X. Peng, *Chem. Mater.* **2003**, *15*, 2854-2860.
[4] E. L. Rosen, R. Buonsanti, A. Llordes, A. M. Sawvel, D. J. Milliron, B. A. Helms, *Angew. Chem. Int. Ed.* **2012**, *51*, 684-689.
[5] Y.-Y. Cai, X.-H. Li, Y.-N. Zhang, X. Wei, K.-X. Wang, J.-S. Chen, *Angew. Chem. Int. Ed.* **2013**, *52*, 11822-11825.
[6] Z. Zheng, T. Tachikawa, T. Majima, *J. Am. Chem. Soc.* **2015**, *137*, 948-957.
[7] Z. Zhang, S.-W. Cao, Y. Liao, C. Xue, *Appl. Catal.: B Environ.* **2015**, *162*, 204-209.
[8] K. Tsutsumi, N. Kashimura, K. Tabata, *Silicon* **2015**, *7*, 43-48.
[9] H. Yoneyama, N. Matsumoto, H. Tamura, *Bull. Chem. Soc. Jpn.* **1986**, *59*, 3302-3304.
[10] Y. Li, F. He, S. Peng, D. Gao, G. Lu, S. Li, *J. Mol. Catal. A: Chem.* **2011**, *341*, 71-76.
[11] X. Zhang, M. Yang, J. Zhao, L. Guo, *Int. J. Hydrogen Energy* **2013**, *38*, 15985-15991.
[12] G. Halasi, G. Schubert, F. Solymosi, *Catal. Lett.* **2012**, *142*, 218-223.
[13] V. Lanese, D. Spasiano, R. Marotta, I. Di Somma, L. Lisi, S. Cimino, R. Andreozzi, *Int. J. Hydrogen Energy* **2013**, *38*, 9644-9654.
[14] B. Loges, A. Boddien, H. Junge, J. R. Noyes, W. Baumann, M. Beller, *Chem. Commun.* **2009**, 4185-4187.
[15] M. Onishi, *J. Mol. Catal.* **1993**, *80*, 145-149.
[16] B. Zielinska, M. Janus, R. Kalenczuk, *Cent. Eur. J. Chem.* **2013**, *11*, 920-926.
[17] A. Boddien, B. Loges, F. Gärtner, C. Torborg, K. Fumino, H. Junge, R. Ludwig, M. Beller, *J. Am. Chem. Soc.* **2010**, *132*, 8924-8934.
[18] I. Willner, Z. Goren, *J. Chem. Soc., Chem. Commun.* **1986**, 172-173.
[19] W. Tang, D. Jing, L. Guo, *MRS Onl. Proc. Libr.* **2011**, *1326*.
[20] S. Kambe, M. Fujii, T. Kawai, S. Kawai, F. Nakahara, *Chem. Phys. Lett.* **1984**, *109*, 105-109.
[21] M. Matsumura, M. Hiramoto, T. Iehara, H. Tsubomura, *J. Phys. Chem.* **1984**, *88*, 248-250.
[22] Y. Li, Y. Hu, S. Peng, G. Lu, S. Li, *J. Phys. Chem. C* **2009**, *113*, 9352-9358.
[23] Y. Li, L. Tang, S. Peng, Z. Li, G. Lu, *CrystEngComm* **2012**, *14*, 6974-6982.
[24] Y. J. Zhang, L. Zhang, *Desalination* **2009**, *249*, 1017-1021.
[25] Y. J. Zhang, L. Zhang, S. Li, *Int. J. Hydrogen Energy* **2010**, *35*, 438-444.
[26] X. Wang, W.-C. Peng, X.-Y. Li, *Int. J. Hydrogen Energy* **2014**, *39*, 13454-13461.
[27] H. M. Yeh, S. L. Lo, M. J. Chen, H. Y. Chen, *Water Sci. Technol.* **2014**, *69*, 1676-1681.
[28] A. I. Nedoluzhko, I. A. Shumilin, V. V. Nikandrov, *J. Phys. Chem.* **1996**, *100*, 17544-17550.
